# Is Online Patient-Centered Information About Implant Bone Graft Valid?

**DOI:** 10.7759/cureus.46263

**Published:** 2023-09-30

**Authors:** Muath S Alassaf, Hassan A Hammudah, Esam S Almuzaini, Ahmad A Othman

**Affiliations:** 1 Orthodontics and Dentofacial Orthopedics, Taibah University, Madina, SAU; 2 Dental Education, Taibah University, Madina, SAU; 3 Dentistry, Taibah University, Madina, SAU

**Keywords:** jama benchmark, dental implant, patient education, web-based knowledge, ridge augmentation, discern

## Abstract

Background

A dental implant is one of the most commonly used treatments to replace missing teeth. A reasonable number of implant cases necessitate using a bone graft before or at the time of implant placement. This study aims to evaluate the quality and readability of online patient-centered information about implant bone grafts.

Methodology

This cross-sectional study used Google, Yahoo, and Bing search engines. The keywords were entered to screen 900 websites. The DISCERN, Journal of the American Medical Association (JAMA), and Health on the Net (HON) code tools evaluated the included websites for quality. The Flesch reading-ease score (FRES), Flesch-Kincaid grade level, and simple measure of gobbledygook tests measured readability. Statistical analysis was done using SPSS version 25 (IBM Corp., Armonk, NY, USA).

Results

A total of 161 websites were included; 65 (40.4%) of the included websites belonged to a university or medical center. Only five (3.1%) websites were exclusively related to dental implant treatments. DISCERN showed moderate quality for 82 (50.9%) websites. There was a statistical difference between commercial and non-profit organization websites. In the JAMA evaluation, currency was the most commonly achieved in 67 (41.6%) websites. For the HON code, four (2.5%) websites were certified. Based on FRES, the most common readability category was “fair difficult,” accounting for 64 (39.8%), followed by “standard” in 56 (34.8%) websites.

Conclusions

The study findings suggest that English-language patient-centered information about implant bone grafts is challenging to comprehend and of low quality. Hence, there is a need to establish websites that provide trustworthy, high-quality information on implant bone grafts.

## Introduction

Tooth loss can be a serious issue affecting millions of people worldwide. In fact, in 2015, a staggering 276 million people experienced complete tooth loss. Poor oral hygiene and careless behavior about oral health can lead to periodontal problems and teeth loss. Recent studies investigating the reasons for teeth extraction found that the most common reason was dental caries, followed by periodontal diseases [1,2]. The number of teeth lost per individual is very high. According to the Centers for Disease Control and Prevention of Oral Health Surveillance in the United States, the average number of teeth remaining in the population aged 20-64 years is 25.3, which means that the average number of extracted teeth is 6.7 per person [1,3].

For replacing missing teeth, people opt for different treatments. The survival rate differs according to the type of treatment. For example, the single crown supported by implant survival rate was 96.363%. On the other hand, it was 94.525% for fixed partial dentures and 91.27% for implant tooth-supported prostheses after five years [4]. Dental implant is among the best choices because of the high survival rate. It has up to 98% survival rate in the first five years and 90-95% after 10 years of follow-up [3,5]. The use of bone grafts can improve the survival rate of implants and aid in the healing of soft and hard tissues [6].

After tooth extraction, biological and physiological bone remodeling occurs. There are some debates about bone resorption and the preservation of alveolar bone with the use of an implant because of the percentage of bone loss around the implant, even if it is low. Until now, there is not enough evidence to conclude whether the implant can preserve the alveolar bone height [3,7]. The amount of bone loss around a dental implant and a natural tooth is not the same. It tends to be minimal in natural teeth but more significant in dental implants. The implant treatment is considered unsuccessful if the bone loss around a dental implant exceeds 1 mm in the first year or 0.2 mm annually after that [8].

Implant placement requires good bone quality and morphology, which includes bone height and width. In cases where bone is deficient, bone graft or ridge augmentation is indicated. There are several types of bone graft materials, each with their specific use and properties [9]. Based on the source of the bone, it is classified into autograft, allograft, xenograft, and alloplastic. Each type has certain properties and different biological mechanisms [8,9]. Different types of bone grafts and different combinations around the implant have different survival rates [10]. This can make this title very wide, and each clinician has their own mix or technique in bone grafting. Patients can also be confused about the term bone graft, and even after explaining the procedure to them, they need more time and information to understand it. Nowadays, almost everyone owns a phone, which provides easy access to the Internet. A study of 520 people showed that 96% used the phone daily, and 34% used the Internet to search for dental information [11]. Internet health information can also affect patient trust and compliance [12]. The three easily accessible search engines worldwide are Google, Yahoo, and Bing [13]. Many researchers have investigated the accuracy of web-based information about health-related topics. In the field of dental implantology, studies have aimed to evaluate the web-based knowledge about implants and peri-implantitis. The studies reported that content is difficult to read and of low quality [14,15]. The available online information varies greatly across different websites, even when searching with the same terms [15]. Currently, no studies have been conducted on the web-based knowledge available about implant bone grafts. This study aims to evaluate the quality and readability of patient-focused information related to bone grafts for implant treatment.

## Materials and methods

The utilization of websites

The technique used for finding websites involved searching and choosing from a common English search engine, such as Google, Yahoo, and Bing. The searches were accomplished in incognito mode to guarantee impartial outcomes, and cookies and browser data were cleared preceding browsing. The search was based on the following keywords: “Dental bone graft” and “Implant bone graft” in the search algorithm. Despite only a small fraction of users typically clicking on results beyond the first page of Google [16,17], we included the first 150 websites from each search engine per term in the study to ensure comprehensive coverage. The default search settings were retained, and advanced search options were not utilized.

We implemented exclusion criteria to eliminate irrelevant websites. Websites with non-English content, those with a minimal amount of information about bone grafts, sites primarily featuring auditory or visual content, scientific publications or books, websites involving banner advertisements or promotional products, sponsored links, or discussion forums, websites restricting direct access, and websites lacking information about ridge augmentation were excluded. Finally, duplication was checked and removed.

The remaining incorporated websites were classified following the framework proposed by Ni Riordain and McCreary in 2009 [18]. They were categorized based on their affiliation as non-profit organizations, university/medical centers, or governmental entities. Additionally, the specialization of the websites was classified as whether they were partially or exclusively related to ridge augmentation. The content types were identified as medical facts, clinical trials, question and answers, and human-interest stories. At the end, websites with content presentation included audio, images, and video.

Quality assessment

The evaluation of the websites was performed using well-established assessment tools, which included the Journal of the American Medical Association (JAMA) [19] criteria for website analysis, the Health on the Net (HON) code [20], and the DISCERN evaluation instrument [21].

The DISCERN questionnaire offers a reliable approach to assess the quality of written information concerning various therapies for specific health conditions. This questionnaire comprises 16 questions categorized into three distinct sections. The first part, encompassing questions 1 to 8, primarily evaluates the publication’s credibility, aiming to find its reliability as a source of information about a particular therapy. The second part consists of questions from 9 to 15, focusing specifically on the quality of provided content about treatment options. Lastly, question 16 corresponds to the overall quality score of the evaluation. The sum of scores was calculated as the total score. Hence, 80 was the maximum, and 16 was the minimum. Reliability and treatment quality scores reflected the sum of questions 1 to 8 and 9 to 15, respectively. A five-point Likert scale was utilized to assign scores to each question, with a rating of one indicating no or poor quality and five signifying good quality [21]. To establish consistency in website evaluations using the DISCERN questionnaire, a dentist with expertise in the field reviewed the uniformity of website ratings. Based on the total score, websites were categorized as follows: low scores (16-32), moderate scores (33-64), and high scores (≥65).

The Journal of American Medical Association has published the JAMA benchmarks, which encompass the following four criteria: authorship (the website clearly states the authors of the medical content, as well as their affiliations and relevant credentials), attribution (sources of its information, including any references or studies), currency (clearly indicating when the medical content was posted or updated), and disclosure (ownership and disclosure of any conflicts of interest). Each item is evaluated as achieved or not [19].

Each website was additionally assessed for the transparency and quality of online health information using the HON (Health On the Net) Foundation. It is worth mentioning that while HON primarily concentrates on evaluating transparency and quality, it does not evaluate the accuracy of online health information. HON includes the following eight criteria: attribution, authority, complementarity, confidentiality, justifiability, financial disclosure, openness, and advertising policy [20]. The evaluation of the HON code was conducted using a browser extension provided on their official website.

Readability assessment

Regarding the readability assessment, three tools were utilized, namely, the Flesch-Kincaid grade level (FKGL), the simplified measure of gobbledygook (SMOG), and the Flesch reading ease (FRE) scale. FKGL ranges from 0 to 18, with 18 being the most difficult text to read. The SMOG readability test gauges the education level required to comprehend a text; the higher the score, the more difficult the text to comprehend, with each score corresponding to education level (7-8 means the text is readable for the 7th to 8th-grade level). However, the FRE score can range from 0 to 100; the higher the score, the easier the text to read. An FRE score of 90-100 means the text is very easy to understand, 80-89 is easy, 70-79 is fairly easy, 60-69 is standard and ideal for most general audience material, 50-59 is fairly difficult to read, and below 50 is difficult to read (graduate level). All readability tests were conducted using the automated formulas for the intended formulas.

Ethical considerations

As this study relies solely on public data, ethical approval and consent were not necessary.

Data analysis

The statistical data was analyzed using the statistical software SPSS version 25 (IBM Corp., Armonk, NY, USA). The results were presented in tables, with each mean value accompanied by its standard deviation. A p-value of less than 0.05 was considered statistically significant for comparative tests.

## Results

Available websites and categorization

**Figure 1:**
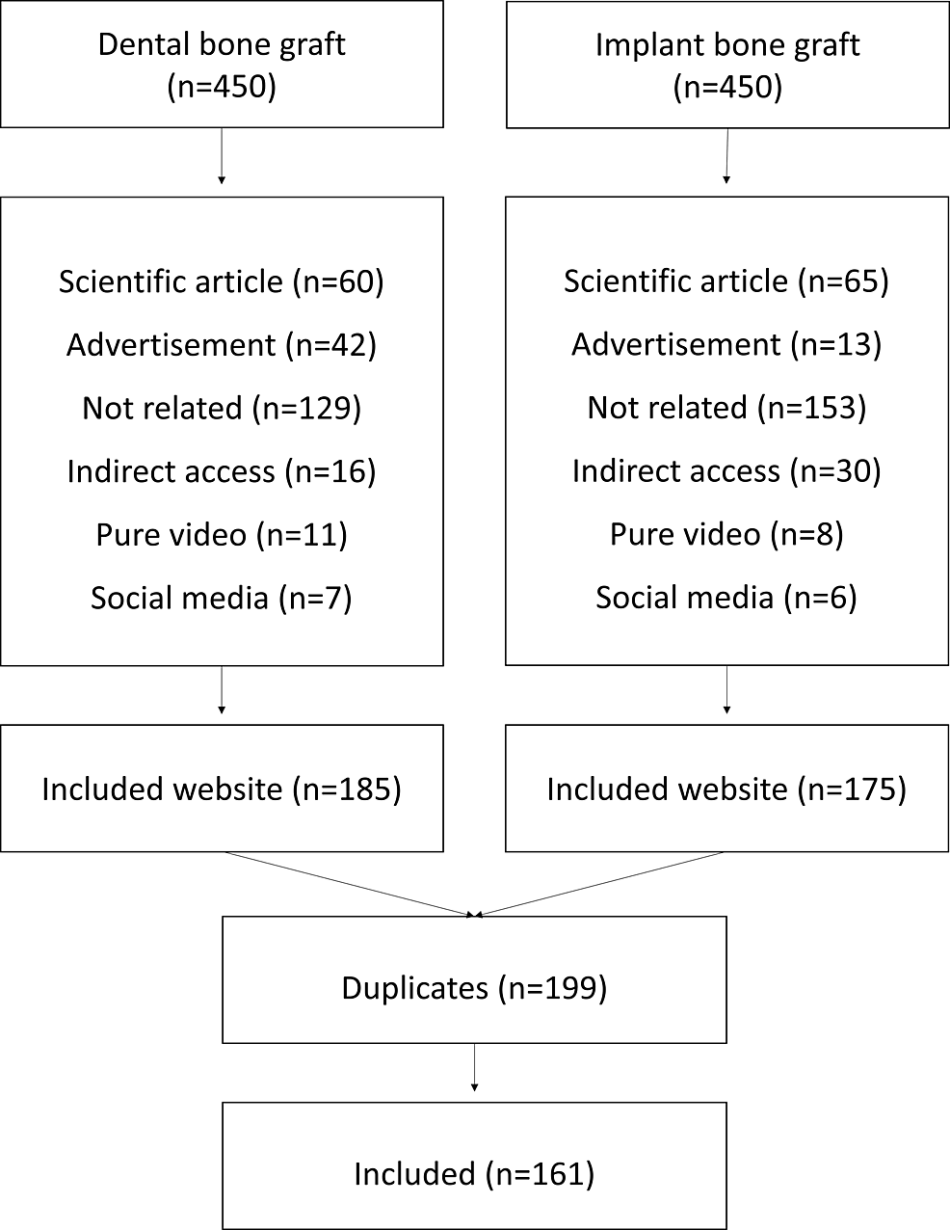
Flowchart of the search strategy and website exclusion.

Searching the two terms “Dental bone graft” and “Implant bone graft” in Google, Bing, and Yahoo resulted in 28,200,000, 757,000, and 861,000 for the term “dental bone graft” per engine, respectively. For “Implant bone graft,” it resulted in 23,000,000 for Google, 821,000 for Bing, and 1,330,000 for Yahoo. The first 150 websites per search term from each search engine were screened across the eligibility criteria. Among the 900 websites, 161 were included. Figure 1 shows the exclusion process. Based on affiliation, 65 (40.4%) of the included websites belonged to a university or medical center. Commercial websites accounted for 47 (29.2%), followed by 33 (20.5%) as non-profit organizations, and only 16 (9.9%) were governmental. Only five (3.1%) websites were exclusively related to dental implant treatments. None of the websites included human interest stories or audio. The content type and presentation with a summary of website categorization are presented in Table 1.

**Table 1:** Websites categories according to affiliation, content type, and presentation of the included websites (n = 161).

Category	Criteria	n (%)
Affiliation	Commercial	47 (29.2)
	Non-profit organization	33 (20.5)
	University/medical center	65 (40.4)
	Governmental	16 (9.9)
Specialization	Exclusively related	5 (3.1)
	Partly related	156 (96.9)
Content type	Medical facts	159 (98.8)
	Clinical trials	1 (0.6)
	Human interest stories	0 (0)
	Question and answer	87 (54)
Content presentation	Image	81 (50.3)
	Video	36 (22.4)
	Audio	0 (0)

Quality assessment

Quality was assessed using DISCERN, JAMA benchmarks, and HON. Total DISCERN had a mean score of 47.97 (±17.62), and the overall quality (question 16) mean was 2.94 (±1.16). The reliability and treatment quality sections had a mean of 23.88 (±8.427) and 21.16 (±8.623), respectively. Table 2 shows the mean scores per DISCEN questions. The poorest quality score was related to the fourth question, “Is it clear what sources of information were used to compile the publication?” In contrast, the highest was related to the first question about explicating aims. Total score quality categorized websites into moderate quality for 82 (50.9%) of the websites as the most common category. Low and high quality accounted for 46 (28.6%) and 33 (20.5%), respectively. The distribution of the quality based on website affiliation is shown in Table 3. No significant difference was found between the DISCERN quality category and affiliation. However, as shown in Table 4, the overall quality and website reliability mean score was statistically significant for commercial websites and websites belonging to non-profit organizations (p < 0.05).

**Table 2:** Mean scores and standard deviation (SD) of DISCERN questions for the included websites (n = 161).

Domain	DISCERN question	Mean (SD)
Reliability	Q1. Explicit aims	3.81 (1.2)
	Q2. Aims achieved	3.37 (1.2)
	Q3. Relevance	3.56 (1.2)
	Q4. Explicit sources	1.68 (1.4)
	Q5. Explicit dates	3.04 (1.7)
	Q6. Balanced and unbiased	2.66 (1.1)
	Q7. Additional sources	2.88 (1.2)
	Q8. Areas of uncertainty	2.88 (1.6)
Treatment options	Q9. How treatment works	3.11 (1.3)
	Q10. Benefits of treatment	3.34 (1.3)
	Q11. Risk of treatment	3.22 (1.5)
	Q12. Effects of no treatment	2.63 (1.2)
	Q13. Effects on quality of life	2.82 (1.6)
	Q14. All alternatives described	3.04 (1.2)
	Q15. Shared decision	2.99 (1.7)
Overall rating	Q16. Overall quality rating	2.94 (1.1)

**Table 3:** Quality and readability of the included websites based on their affiliation reported as frequency and percentage. * Statistically significant differences at a level of 0.05 or less. JAMA: Journal of the American Medical Association

Variable	Commercial	Governmental	Non-profit organization	University/Medical Center	Total	P-value
Number of achieved JAMA items per website						
None	23 (14.3%)	8 (5.0%)	12 (7.5%)	34 (21.1%)	77 (47.8%)	0.62
One	11 (6.8%)	3 (1.9%)	6 (3.7%)	13 (8.1%)	33 (20.5%)	
Two	9 (5.6%)	3 (1.9%)	6 (3.7%)	12 (7.5%)	30 (18.6%)	
Three	4 (2.5%)	2 (1.2%)	9 (5.6%)	6 (3.7%)	21 (13.0%)	
JAMA items and HON code						
Authorship	16 (9.9%)	5 (3.1%)	16 (9.9%)	24 (14.9%)	61 (37.9%)	0.53
Attribution	4 (2.5%)	3 (1.9%)	13 (8.1%)	8 (5.0%)	28 (17.4%)	0.02*
Currency	21 (13.0%)	7 (4.3%)	16 (9.9%)	23 (14.3%)	67 (41.6%)	0.59
HON Code	0 (0.0%)	1 (0.6%)	3 (1.9%)	0 (0.0%)	4 (2.5%)	0.021*
DISCERN categories based on total evaluation score						
Low	17 (10.6%)	6 (3.7%)	5 (3.1%)	18 (11.2%)	46 (28.6%)	0.08
Moderate	26 (16.1%)	7 (4.3%)	16 (9.9%)	33 (20.5%)	82 (50.9%)	
High	4 (2.5%)	3 (1.9%)	12 (7.5%)	14 (8.7%)	33 (20.5%)	

**Table 4:** Comparison between means for DISCERN and readability scores of the included websites based on their affiliation. *: Statistical significance between commercial and non-profit organizations. FRES: Flesch reading-ease score; FKGL: Flesch-Kincaid grade level; SMOG: simple measure of gobbledygook

Variable	Commercial	Governmental	Non-profit organization	University/Medical Center	P-value
DISCERN					
Overall	43.83 (16.584)	44.25 (18.774)	53.82 (16.855)	48.91 (17.869)	0.041*
Reliability	21.47 (7.535)	22.56 (8.989)	27.09 (8.424)	24.31 (8.491)	0.017*
Treatment	19.68 (8.511)	18.94 (8.978)	23.36 (8.146)	21.65 (8.728)	0.21
Readability					
FRES	57.29 (10.6033)	55.388 (8.8684)	57.564 (7.2666)	56.323 (8.9637)	0.68
FKGL	9.906 (2.1292)	10.2 (1.8037)	9.912 (2.0324)	10.189 (1.9822)	0.59
SMOG	9.149 (1.682)	9.462 (1.5392)	9.221 (1.2173)	9.32 (1.4646)	0.63
Words	948.6 (530.402)	1,106.38 (469.461)	1,147.76 (557.772)	1,129.54 (667.475)	0.26
Sentences	52.85 (34.831)	60.56 (29.864)	64.21 (33.572)	61.25 (36.905)	0.21

Regarding JAMA benchmarks, none of the websites achieved the four items, while three items were achieved in 21 (13%) websites; nine of these websites belonged to non-profit organizations. No significant difference was observed between the number of achieved items among different affiliations (p = 0.62). JAMA evaluation includes four items. Currency was the most commonly achieved in 67 (41.6%), followed by authorship in 61 (37.9%) websites. Attribution was met in 28 (17.4%) websites only, with 13 (8.1%) belonging to non-profit organizations with statistically significant differences (p = 0.02). None of the websites achieved the disclosure item. The summary of the JAMA evaluation among affiliations is shown in Table 3. For the HON code, four (2.5%) websites were certified. Three (1.9%) websites belonged to non-profit organizations with statistical differences (p = 0.021).

Readability

**Figure 2:**
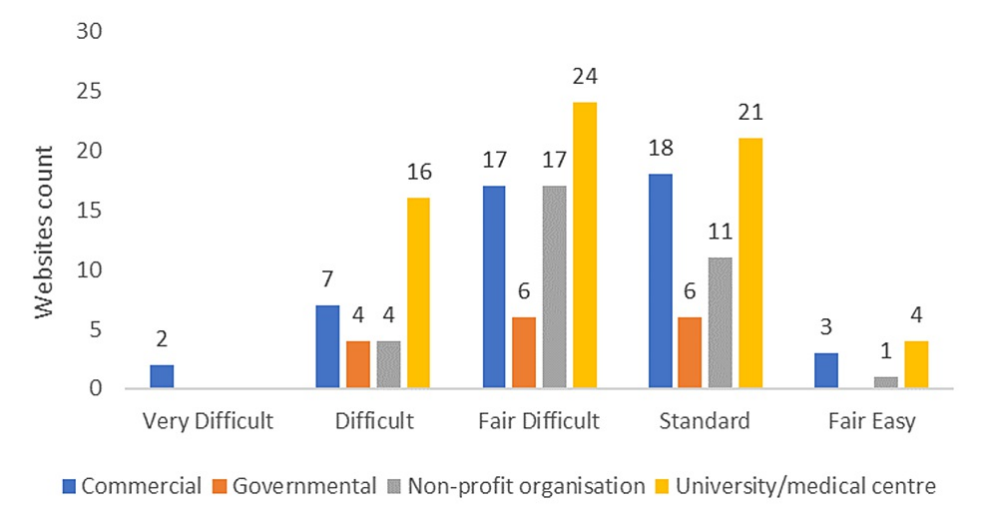
Flesch reading-ease score readability categories according to website affiliation (n = 161).

FRE scale ranged from 19.7 to 78.5 with a mean of 56.76 (±9.1), and the most common category was “fair difficult,” accounting for 64 (39.8%), followed by “standard” in 56 (34.8%) websites. Only eight (5%) websites were “fair easy”. Figure 2 shows the distribution of FRE scale categories based on the websites’ affiliations. FKGL varied from 5.9 to 18.5 with a mean of 10.1 (±2). SMOG had a minimum score of 6.3 and a maximum score of 14.1, with a mean score of 9.1 (±1.5). As shown in Table 4, no statistical differences were found between the mean readability scores across affiliations.

Word and sentence count varied among websites, ranging from 171 to 3,365 with a mean of 1,078.2 (±590.8) and 7 to 227 with a mean of 59.3 (±34.9), respectively. Different means according to affiliation are shown in Table 4.

## Discussion

The internet has improved many aspects of life, including access to healthcare through telemedicine. While it can be helpful in reducing health illiteracy, there are risks of inaccurate information and incomplete knowledge delivery [22]. With the internet becoming a major source of health information, it is essential to have accurate, trustworthy, and easy-to-read content to avoid negative effects caused by misinformation. In a study on oral health, 56.1% of participants utilized the Internet to gather information about their children’s conditions [23]. Evaluating online health information is important for patients and practitioners to determine its reliability and safety in guiding patient decisions.

A dental implant is the first and most reliable choice for replacing missing teeth, and more than half of the implant cases require bone graft [24]. The assessment of patient-centered knowledge regarding dental implants and their complications has been completed. However, it is imperative that the online bone graft content undergoes an evaluation as well [14,15]. To evaluate the quality of medical websites, tools such as JAMA benchmarks, DISCERN, and HON code, were created and extensively used for different diseases and treatments.

Content types and presentation

After filtering the 900 screened websites for Google, Bing, and Yahoo, only 161 websites were included, with the most common reason for exclusion being that the website is not related. The studies about dental implants and peri-implantitis included 32 and 27 websites, respectively [14,15]. This difference is likely due to their use of two search engines and screening only the first 100 websites. The number of screened websites was expanded to ensure a comprehensive evaluation.

Based on website affiliations, most websites belonged to universities or medical centers, followed by commercial websites. This is more or less consistent with the other studies, as the study about implants had non-profit organizations and university or medical centers as the most common. However, in the peri-implantitis study, it was commercial and non-profit organizations. Noteworthy, governmental websites were the least in both and in this study [14,15]. Governmental patient information was evaluated before and showed that more than 75% were complicated for the general population [25]. Regarding website specialization, over 90% of the websites in this study were partly related, which is also consistent with the other studies [14,15]. In similar studies, the content type was mostly medical facts, and this was true even for non-implant-related topics [26,27]. In our studies, websites containing questions and answers were more than half of the websites compared to only 10% in the study about peri-implantitis. This is likely due to the difference in the number of evaluated websites [15]. The content presentation affects perception and understanding, and videos were found to be more effective [25]. In this study, about half of the websites included images, and 22% contained videos.

Quality assessment

DISCERN evaluates two aspects, namely, the reliability of the information and the quality of content about the treatment. Among the 16 questions, the poorest scores were related to the references. The mean score for question four was 1.68 (±1.4), lower than the studies in different fields [14,15,28]. The overall quality mean was 47.97 (±17.62) and was considered similar to multiple studies. The total mean score for DISCERN was higher than in commercial websites with statistical differences (p = 0.041). The primary challenges associated with the DISCERN tool stemmed from the absence or inadequate availability of information sources and a lack of information about how treatment works, risks, supplementary therapies, and potential alternatives in the context of ridge augmentation. The mean for the reliability section was also higher in non-profit organizations. The section about treatment information did not differ significantly. Therefore, commercial and non-profit organization websites have a similar quality, but non-profit organizations’ websites are more reliable. For the DISCERN categories, moderate scores were the most common, and high scores were mostly in universities or medical centers. These findings are consistent with other studies, but the percentage of high-quality websites was higher for websites in this study [26,29].

JAMA evaluation showed similar findings to other studies in terms of the number of achieved items per website, with most websites not achieving any item. Our data showed a statistically significant difference in attribution among different affiliations, with non-profit organizations being higher in including sources of information.

Websites that adhere to the HON code are expected to provide accurate, transparent, and up-to-date information; protect user privacy; and clearly disclose the source and qualifications of the content creators [20]. Similar studies about the number of websites with HON seal rangers from 4-12 websites [14,15,26]. In this study, there were four websites, with three belonging to non-profit organizations. All websites presenting information to patients should be encouraged to obtain the HON code to provide patients with an easy way to know what websites they can rely on when looking for health-related information.

Readability assessment

Low health literacy has been found to have a negative impact on health and quality of life. Research has shown that older adults with low health literacy are more likely to experience chronic health conditions and have a higher risk of mortality [30]. Population-targeting knowledge should be presented in simple and understandable terms so non-medical individuals can comprehend the information. Common readability tests use the number of words and sentences with the number of difficult words to calculate the readability of a given text. Standard well-established readability tests include FRES, FKGL, and SMOG [19]. This study showed comparable means for readability tests among different affiliations. However, it shows that about a hundred websites were in the very difficult, difficult, and fair difficult range. Only about one-fourth of the websites had a standard readability. Compared to other studies, these findings are in line with the literature, which indicates a need for improvement of patient-centered knowledge [14,15,19,26,30]. These improvements can be accomplished by avoiding medical jargon and low-frequency vocabulary. Moreover, this situation is particularly worrying because a large number of people have limited health literacy. This means they struggle to access, understand, evaluate, and communicate health-related information [30].

Limitations

It is important to acknowledge the limitations of our study. We only searched three search engines. Additionally, we only used six assessment tools for quality and readability. Although they are commonly used, we encourage further research that addresses these limitations. In addition, our study evaluated the content in the English language only.

## Conclusions

The study findings suggest that English-language patient-centered information about implant bone grafts is challenging to comprehend and lacks good quality. This can potentially contribute to the spread of misinformation and the persistence of unrealistic patient expectations. As a result, there is a need to establish websites that provide trustworthy, high-quality information on implant bone grafts. Such resources should be free from commercial biases and presented in an easily understandable way for the average patient.
